# EGCG Nanoparticles Attenuate Aluminum Chloride Induced Neurobehavioral Deficits, Beta Amyloid and Tau Pathology in a Rat Model of Alzheimer’s Disease

**DOI:** 10.3389/fnagi.2018.00244

**Published:** 2018-08-13

**Authors:** Neha Atulkumar Singh, Vaishali Bhardwaj, Chandrika Ravi, Nithya Ramesh, Abul Kalam Azad Mandal, Zaved Ahmed Khan

**Affiliations:** ^1^Department of Integrative Biology, School of Biosciences and Technology, Vellore Institute of Technology, Vellore, India; ^2^Department of Biotechnology, School of Biosciences and Technology, Vellore Institute of Technology, Vellore, India; ^3^University Institute of Biotechnology, Chandigarh University, Mohali, India

**Keywords:** Alzheimer disease, aluminum chloride, EGCG, nanoparticles, neurobehavioral impairments, neuritic plaques, neurofibrillary tangles

## Abstract

**Rational**: Alzheimer’s disease (AD) is a neurodegenerative pathology characterized by the presence of neuritic plaques and neurofibrillary tangles. Aluminum has been reported to play an important role in the etiology and pathogenesis of this disease. Hence, the present study aimed to evaluate the neuroprotective role of epigallocatechin-gallate (EGCG) loaded nanoparticles (nanoEGCG) against aluminum chloride (AlCl_3_) induced neurobehavioral and pathological changes in AD induced rats.

**Method**: 100 mg/kg body weight AlCl_3_ was administered orally for 60 days, which was followed by 10 mg/kg body weight free EGCG and nanoEGCG treatment for 30 days. Morris water maze, open field and novel object recognition tests were employed for neurobehavioral assessment of the rats. This was followed by histopathological assessment of the cortex and the hippocampus in the rat brain. For further validation biochemical, immunohistochemistry and western blot assays were carried out.

**Result**: Aluminum exposure reduced the exploratory and locomotor activities in open field and significantly reduced the memory and learning curve of rats in Morris water maze and novel object recognition tests. These neurobehavioral impairments were significantly attenuated in nanoEGCG treated rats. Histopathological assessment of the cortex and hippocampus of AlCl_3_ induced rat brains showed the presence of both neuritic plaques and neurofibrillary tangles. In nanoEGCG treated rats this pathology was absent. Significant increase in biochemical, immunohistochemical and protein levels was noted in AlCl_3_ induced rats. While these levels were greatly reduced in nanoEGCG treated rats.

**Conclusion**: In conclusion, this study strengthens the hypothesis that EGCG nanoparticles can reverse memory loss, neuritic plaque and neurofibrillary tangles formation.

## Background

Alzheimer’s disease (AD) is a progressive neurodegenerative disorder that imposes significant economic and social burden on society (Singh et al., 2016). AD is mainly classified into two forms; sporadic and non-sporadic, where less than 5% cases account for the non-sporadic form and 95% cases account for the sporadic form. The familial or non-sporadic form of AD is early onset, typically occurs in individuals younger than 65 years. The predominance of AD doubles every 5 years, after the age of 65 in the late-onset sporadic form of AD (Thal and Fändrich, 2015). AD is the most common form of dementia associated with neuropathological and neurobehavioral changes, where the symptoms gradually worsen over the years (Cam and Bu, 2006). AD can be classified into three main stages based on the progression of the disease namely, mild, moderate and severe. The first clinical symptom of AD is memory impairment, specifically short-term memories, whereas the long-term memories are well preserved. With disease progression impairment in cognitive abilities becomes visible, executive decision-making and ability to carry out daily chores diminish significantly (Kimura and Ohno, 2009). The pathological hallmarks of AD include positive lesions such as amyloid plaques, neurofibrillary tangles, cerebral amyloid angiopathy and glial responses along with negative lesions such as synaptic loss and neuronal loss. The most important biomarkers for AD pathology include phosphorylated tau protein (P-tau) and 1–42 amino acid form of beta amyloid (Aβ_42_). Of these Aβ_42_ fibril accumulation is considered to be the instigator of this neurodegenerative pathology and a cascade of events including neurotoxicity, inflammation and oxidative stress (Hardy and Selkoe, 2002). The primary cause of death in AD patients is not usually due to these changes in the brain but due to their associated complications such as immobility, pneumonia and malnutrition induced due to trouble in swallowing (Jönsson et al., 2006). Patients are affected by AD differently, as their experience in symptoms and disease progression is different (Albert et al., 2011). This is mainly due to the difference in factors such as genetics, age, education and co-morbidities (Kim et al., 2002).

Aluminum is a well-established neurotoxicant involved in the etiology of AD (Sun et al., 2009). It is an abundant metal on earth with easy access to the human body through antacids, water, food additives, utensils, deodorants and drugs. In the brain, aluminum predominantly accumulates in the hippocampus and frontal cortex, regions known to be particularly susceptible in AD (Flaten, 2001; Campbell, 2002). It induces misfolding of cytoskeleton proteins which leads to the formation of amyloid beta plaques (Campbell et al., 2000; Kawahara et al., 2001) and tau neurofibrillary tangles (El-Sebae et al., 1993) in the brain. Aluminum supplementation causes neurodegeneration and apoptotic neuronal loss (Ghribi et al., 2001), along with cognitive dysfunction, as it is a potent cholinotoxin (Gulya et al., 1990). Normally, cholinergic activity is necessary for the acquisition and retrieval of learning and memory skills (Domingo, 2006). Hence patients with AD normally demonstrate impaired performance in various cognitive tasks. Various animal studies have also shown that prolonged exposure to aluminum can cause neurochemical, neurobehavioral and neuropathological changes in the brain, which impair the learning ability of the rats (Miu et al., 2003; Kaur and Gill, 2006; Prema et al., 2017).

So far there is no effective cure for AD. Current pharmacological treatments only provide temporary symptomatic relief or improve cognitive functions with multiple side effects, but they cannot stop the progression of the disease. Therefore, there is a need to develop a class of drug capable of targeting a broader spectrum of targets to treat not only symptoms but also reverse the pathology of AD with fewer side effects. In the last decade, green tea polyphenols particularly its active component epigallocatechin-gallate (EGCG) has gained a lot of attention as a potential therapeutic agent for AD. Epidemiological studies with EGCG indicate towards a possible relation between AD prevention and the intake of polyphenol rich diet. In addition to their antioxidant and metal chelation properties, they are known to modulate pathways implicated in the pathogenesis of Aβ (Singh et al., 2016). Due to its poor bioavailability and stability its therapeutic potential has been limited. We have resolved these issues via the encapsulation of the EGCG into Poly D, L-lactic acid (PLA); polyethylene glycol (PEG) polymer nanoparticles (nanoEGCG) which has not only enhanced its antioxidant and metal chelation properties but also its ability to produce an inhibitory effect on Al (III) induced Aβ_42_ fibrillation *in vitro*. NanoEGCG was also able to protect cells (*in vitro)* from damage associated with Aβ and notably increase its neuroprotective action, by preventing the production of toxic and insoluble Aβ_42_ fibrils through the generation of soluble and less toxic amorphous aggregates (Singh et al., 2018). Further, PLA-PEG were used over liposomes, lipid or gold nanoparticles because of their versatility, high bioavailability, high encapsulation efficiency, controlled release properties and their outer surface can be easily modified to target specific cells or cross the blood brain barrier (Granja et al., 2017).

Therefore, the present study investigates the neuroprotective action of nanoEGCG in aluminum chloride (AlCl_3_) induced Alzheimeric rats (*in vivo*) by exploring the neurobehavioral cognition, neuropathological and molecular changes in the hippocampus and cortex of Alzheimeric rats. So far there is no report on the use nanoEGCG to treat AD *in vivo*.

## Materials and Methods

### Materials

PLA, PEG, polyvinyl alcohol (PVA), EGCG (>95%), AlCl_3_—hydrated (AlCl_3_.6H_2_O), mouse anti-APP, mouse anti-Aβ_1–42_, rabbit anti-Acetylcholinesterase (AChE), rabbit anti-PDK1, rabbit anti-GSK3β, goat anti-rabbit IgG horse-radish peroxidase (HRP) conjugated, goat anti-mouse IgG HRP conjugated, dichlorodihydrofluorescein diacetate (H2DCFDA) and 5,5′-dithiobis-(2-nitrobenzoic acid; DTNB) were purchased from Sigma Aldrich, St Louis, MO, USA. All other chemicals used were of analytical grade.

### Synthesis and Characterization of NanoEGCG

In our previous study, we have synthesized nanoEGCG using double emulsion solvent evaporation method. Briefly, 0.1% PVA—EGCG and PLA—PEG—DCM solutions were prepared and mixed together via sonication for 30 s to in order to generate a water—oil emulsion. 0.7% PVA solution was then added to this mixture and sonicated for 1 min to generate a water—oil—water emulsion. The organic solvent was evaporated at room temperature and the nanoparticles were recovered by centrifugation at 12,000 *g* at 4°C for 20 min. The recovered nanoparticles were washed twice via centrifugation to remove all traces of the surfactant. Before analysis the formulation was sonicated for 1 min to disperse and deagglomerate the nanoparticles. The surface morphology of nanoEGCG was analyzed using transmission electron microscopy (HR-TEM; FEI-Tecnai G2-20 TWIN) and high-resolution scanning electron microscopy (HR-SEM; FEI Quanta FEG 200), while the hydrodynamic diameter and the zeta potential of nanoEGCG (10 mg/mL) was determined using dynamic light scattering (Zetasizer with Ver. 6.12 software). The encapsulation efficiency of nanoEGCG was also determined (Singh et al., 2018).

### Animals

Male Swiss Albino Wistar rats weighing 200–250 g were procured from the animal house, VIT, Vellore, Tamil Nadu. The rats were housed in polypropylene cages and maintained at 12 h light and 12 h dark cycle at 25 ± 3°C temperature with 30%–60% humidity in a well-ventilated room. Standard pellet feed and tap water was provided *ad libitum*. For one week, the animals were acclimatized to these conditions. All experiments were carried out in the light phase between 09.00 and 17.00 h. This study was carried out in accordance with the principles of the National guidelines and were approved by the Institutional animal ethics committee, VIT University, Vellore.

### Experimental Design

**Figure 1:**
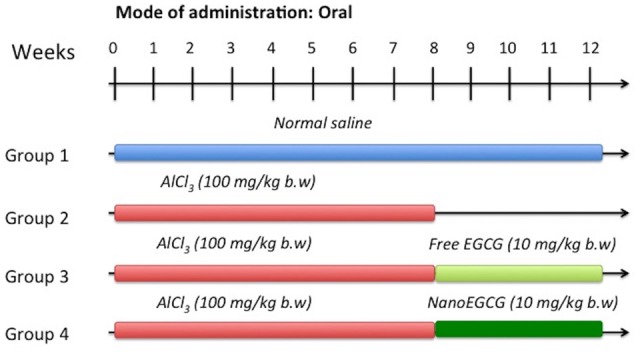
Experiment strategy for aluminum chloride (AlCl_3_) induction, free epigallocatechin-gallate (EGCG) and EGCG nanoparticle (nanoEGCG) treatment in wistar rats.

Forty randomly selected rats were divided into four groups containing 10 animals each (Figure 1).

Group I: Rats treated with normal saline by oral gavage.
Group II: Rats induced with AlCl_3_ (100 mg/kg/body weight) for 60 days by oral gavage (Nampoothiri et al., 2015).
Group III: Rats induced with AlCl_3_ as group II and subsequently treated with free EGCG (10 mg/kg/body weight) for 30 days by oral gavage.
Group IV: Rats induced with AlCl_3_ as group II and subsequently treated with nanoEGCG (10 mg/kg/body weight) for 30 days by oral gavage.

After 24 h of the administration of the last dose, the rats were subjected to multiple behavioral studies. Subsequently they were sacrificed by euthanasia. Blood and brain tissues (cortex and hippocampus) were extracted for further studies.

### Neurobehavioral Studies

#### Open Field

Open field activity monitoring test was performed to assess the locomotor and behavioral activity of the rats. Observations are recorded in a wooden apparatus that is divided into 16 (4 × 4) squares. The animal is placed in one corner of the chamber and its behavior is observed for 5 min. The test measures: (i) squares explored (number); (ii) total time immobile (s); (iii) rearing (number); and (iv) fecal pellets (number; Rajasankar et al., 2009).

#### Morris Water Maze

Morris water maze was performed to assess the retention of working (reference) and spatial memory in rats. The apparatus consists of a large circular pool divided into four quadrants with a platform placed in the forth quadrant. External cues were placed around the pool and they remained unchanged throughout the experiment. These cues served as reference memory. During the acquisition phase, the platform was placed 1 cm above the water level and the rats were subjected to trials for five consecutive days, where the animal was placed in the pool in one of the quadrants, while facing the wall of the pool. The drop location changed for each trial. The animal was allowed to locate the platform and was guided towards the platform if it failed in locating the platform with 120 s. The animal was then allowed to stay on the platform for 20 s (Bhalla et al., 2010). The retrieval test was performed in two phases for both working and spatial memories (Morris, 1984). For spatial memory, the time taken (s) by the rat to reach the platform from immersion into the pool was recorded. While for working (reference) memory, the time spent (s) by the rat in the target quadrant (maximum of 120 s) without the platform was recorded.

#### Novel Object Recognition

Novel object recognition was performed to evaluate hippocampal function and recognition memory specifically working and spatial memory in rats. The apparatus is an open field chamber divided into arenas. During the acquisition phase, the animal is placed in the center of the field with two identical objects placed in two corners of the field. The animal was allowed to explore the field for 10 min. During the retrieval phase, the animal is once again placed in the field with two objects, one familiar (identical object from the acquisition phase) and one novel (one that the animal has never seen before). The animal was allowed to explore the field for 10 min. The time spent (s) exploring the familiar and novel objects is recorded, to calculate the discrimination and recognition index (Antunes and Biala, 2012).

### Histopathological Examinations

Control and experimental rats were sacrificed and the cortex and hippocampus sections were isolated and washed with cold phosphate-buffered saline followed by fixation using 10% formalin. Paraffin-embedded blocks were prepared for both tissues. After sectioning, the paraffin sections were stained with hematoxylin and eosin and examined using a light microscope (Erazi et al., 2010). The experiment was performed in triplicates. Blinded experimenters performed the histopathological rating.

### Immunohistochemistry

Cortex and hippocampus tissues were sectioned using a cryostat. Sections were first deparaffinized using xylene, xylene: ethanol (100%), ethanol (100%), ethanol (95%), ethanol (70%) and ethanol (50%) for 3 min at each step. After deparaffinization, antigen retrieval was carried out by placing the slides in Tris-EDTA buffer (pH 9.0) in a water bath at 100°C temperature. The slides were washed with TBS containing 0.025% triton 100X with gentle agitation. The sections were then blocked in 10% normal serum with 1% BSA in TBS and incubated for 2 h for room temperature. Sections were then incubated in primary antibody; anti-mouse beta amyloid 1–42, clone MM26-2.1.3, monoclonal antibody (1:500) in TBS with 1% BSA overnight at 4°C. After washing with TBS containing 0.025% triton 100X with gentle agitation the sections were incubated with anti-mouse HRP IgG conjugated antibody (1:40,000) in TBS with 1% BSA for 2 h at room temperature. Immunoreactivity was visualized after incubation with DAB for 10 min at room temperature followed by hematoxylin staining for 10 min and dehydration in the alcohol series using an Olympus microscope at high magnification. The experiment was performed in triplicates. Blinded experimenters performed the immunohistochemical rating.

### Acetylcholinesterase Assay

AChE was used as a marker for cholinergic neuron loss in the brain. The AChE activity was measured using Ellman method (Ellman et al., 1961). Briefly, 0.05 ml of protein sample was added to 3 ml of 0.1 M sodium phosphate buffer (pH 8.0). 0.1 ml of acetylthiocholine iodide and 0.1 ml DTNB was added to the mixture. The change in absorbance was measured at 412 nm for 2 min at 30 s intervals using a U.V. spectrophotometer.

### Biochemical Markers of Oxidative Stress

#### Reactive Oxygen Species Generation

Reactive oxygen species (ROS) generation was studied as a marker for oxidative stress in the brain. The ROS activity levels were measured using a previously described protocol with slight modifications (Sharma et al., 2013). Briefly, 0.4 ml of protein sample was dissolved in sodium phosphate buffer (pH 7.2). Two microliter of 2 μM H2DCFDA was added to the sample. Fluorescence intensity was quantified after 30 min of incubation in dark using a fluorospectrophotometer (excitation—499 nm and emission—520 nm).

#### Nitric Oxide Species Generation

Nitric oxide (NO) generation was studied as a marker for oxidative stress in the brain. The NO activity levels were measured using Greiss reagent, which constitutes of 0.2% naphthylethylenediamine, 2% sulphanilamide and 5% phosphoric acid (Green et al., 1982). Briefly, standard sodium nitrate curve was prepared using different concentrations. Equal volumes of protein sample and Greiss reagent were mixed and incubated in dark for 10 min. Following which the absorbance of the mixture was taken at 540 nm using a U.V. spectrophotometer.

#### Protein Level Analysis

Cortex and hippocampus tissues were homogenized in ice-cold RIPA buffer containing 5 μl protease inhibitor. The homogenate was then centrifuged at 12,000 rpm for 15 min at 4°C and the supernatant was separated and quantified for protein concentration using Lowry’s method (Lowry et al., 1951). Fifty microgram of the total protein was loaded and separated on 10% and 16% SDS gels through SDS page depending on the protein of interest’s molecular weight. Once separated the gel was transferred onto a PVDF membrane for 16 h at 4°C using a western blot apparatus. Once transferred the membrane was incubated in 5% blocking solution (5% BSA in Tris buffered saline—Tween 20 solution, TBST) for 8 h at 4°C with gentle agitation. This was done to prevent non-specific binding. After blocking the membrane was incubated with the primary antibody prepared in 5% BSA in TBST. The proteins of interest studied were β actin (mouse monoclonal; 1:5,000) APP (mouse polyclonal; 1:500), Aβ_1–42_ (mouse monoclonal; 1:500), AChE (rabbit polyclonal; 1:1,500), PDK1 (rabbit polyclonal; 1:10,000) and GSK3β (rabbit polyclonal; 1:3,000). After primary antibody incubation the membrane was washed thrice with TBST for 15 min and then incubated with their corresponding secondary antibodies (anti-mouse or anti-rabbit HRP IgG conjugated antibody; 1:40,000) for 2 h at room temperature. Once again after incubation the membrane was washed thrice with TBST for 15 min. Chemiluminescence based technique was employed to visualize the immunoreactive protein. ImageJ analysis software was used to perform densitometric analysis of the protein bands. Data correction was performed by background subtraction and normalization with β actin as internal control.

#### Bioavailability Evaluation

Rats were allowed to fast for 16 h prior to EGCG administration to assess its bioavailability in the plasma and brain. Free EGCG was administrated with an oral gavage at a dose of 10 mg/kg body weight and nanoEGCG was administered at a dose of 10 mg/kg body weight. At pre-determined time intervals of 1, 2, 3, 4, 5, 6, 8 and 24 h the rats were sacrificed and their blood was collected by cardiac puncture and brain tissues were extracted. Blood samples were centrifuged at 16,000 *g* for 5 min and the plasma was aliquoted. Brain tissues were homogenized with sodium phosphate buffer (pH 6.5) and centrifuged at 10,000 *g* for 10 min the supernatant was collected. Ascorbic acid and (AA) and Tris (2-carboxyethyl) phosphine (TCEP) were used as stabilizing agents for EGCG. The samples were snap frozen and stored at −20°C. Unconjugated EGCG was estimated from the samples. For the extraction of unconjugated EGCG, 10 μl of K_2_HPO_4_ was added to the sample and incubated for 45 min at 37°C. Ethyl acetate was used for the extraction of EGCG via the evaporation of the ethyl actetate layer using a nitrogen evaporator. The residue was reconstituted using 100 μl of 15% v/v aqueous acetonitrile solution. Finally, 50 μl of the sample was injected into the HPLC column and quantified using the HPLC/UV method (Dube et al., 2011).

### Statistical Analysis

Statistical analysis was performed with one-way analysis of variance (ANOVA) followed by Tukey’s *post hoc* test using GraphPad Prism trial version 5.0. The experimental data was represented as mean ± SD with 10 rats in each group. The results were considered significant when *p* < 0.001, *p* < 0.01 or *p* < 0.05.

## Results

### Effect of NanoEGCG on the Neurobehavioral Parameters in Alzheimeric Rats

#### NanoEGCG Enhanced Locomotor Activity in Alzheimeric Rats

**Figure 2:**
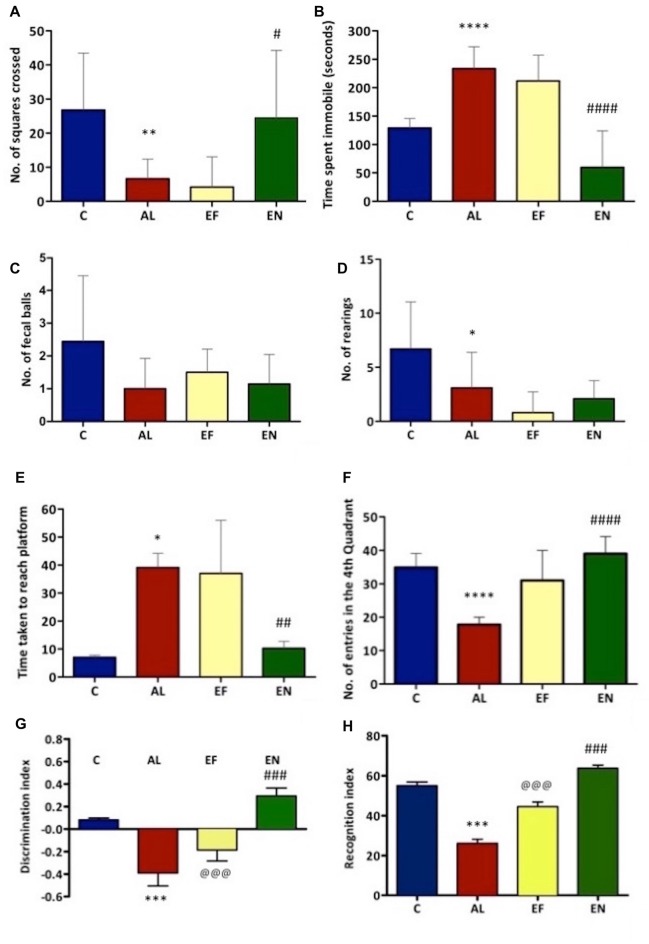
Effect of AlCl_3_ induction, free EGCG and nanoEGCG treatment on **(A)** Open field test—squares explored, **(B)** total time immobile, **(C)** fecal pellets, **(D)** rearing, **(E)** Morris water maze—the time taken to escape to the platform, **(F)** time spent in the target quadrant, **(G)** Novel object recognition test—discrimination index and **(H)** recognition index. Abbreviations: C, control; AL, AlCl_3_ induced Alzheimeric rats; EF, free EGCG treated AlCl_3_ induced Alzheimeric rats; EN, nanoEGCG treated AlCl_3_ induced Alzheimeric rats. Significance difference between groups indicated by: * between C and AL, ^@^ between AL and EF and ^#^ between AL and EN. Significance levels of *p* < 0.0001, *p* < 0.001, *p* < 0.01 or *p* < 0.05 are denoted by repetition of these symbols.

Locomotion and other exploratory behaviors of rats were studied through open field test (Figures 2A,B). A significant (*p* < 0.01) reduction in locomotion was noted in AlCl_3_ induced rats (Alzheimeric rats) with a significant (*p* < 0.0001) increase in time spent immobile as compared to control rats. However, on treatment of these Alzheimeric rats with nanoEGCG a significant (*p* < 0.05) increase in locomotor activity and a significant (*p* < 0.0001) decrease in time spent immobile was noted. Further, no differences were seen between the Alzheimeric rats and the free EGCG treated Alzheimeric rats and between control rats and nanoEGCG treated Alzheimeric rats. In addition, rearing counts and number of fecal balls were also noted, however no significant difference was found between the groups (Figures 2C,D).

#### NanoEGCG Reversed Spatial and Working Memory Deficits in Alzheimeric Rats

Morris water maze was performed to evaluate the retention of working (reference) and spatial memory in Alzheimeric rats. In case of spatial memory, time taken to reach the platform was evaluated. Here, Alzheimeric rats produced a significant (*p* < 0.05) increase in time taken to reach the platform when compared to the control rats. However, treatment of these Alzheimeric rats with nanoEGCG significantly (*p* < 0.01) decreased the time taken to reach the platform, suggesting that nanoEGCG treatment could reverse the spatial memory deficits in Alzheimeric rats (Figure 2E). In case of working (reference) memory, the number of entries in the quadrant where the platform was previously placed was evaluated. Here, Alzheimeric rats showed a significant (*p* < 0.0001) decrease in the number of entries in the target quadrant when compared to the control rats. On the other hand, treatment of these Alzheimeric rats with nanoEGCG significantly (*p* < 0.0001) increased the number of entries in the target quadrant (Figure 2F). Further, no differences were seen between the Alzheimeric rats and the free EGCG treated Alzheimeric rats and between control rats and nanoEGCG treated Alzheimeric rats.

#### NanoEGCG Heightened the Recognition Memory in Alzheimeric Rats

Novel object recognition was performed to evaluate hippocampal function and recognition memory in Alzheimeric rats. The discrimination index analysis revealed that Alzheimeric rats performed very poorly as they were unable to significantly (*p* < 0.001) distinguish between the familiar and novel object when compared to the control rats. However, treatment of these Alzheimeric rats with nanoEGCG showed that they were able to significantly (*p* < 0.001) distinguish between the familiar and novel object when compared to the Alzheimeric rats (Figure 2G). In addition, the recognition index analysis revealed that Alzheimeric rats were unable to significantly (*p* < 0.001) recognize the novel object when compared to the control rats. While on the treatment of these Alzheimeric rats with nanoEGCG showed that they were able to significantly (*p* < 0.001) recognize the novel object when compared to the Alzheimeric rats (Figure 2H). Further, significant (*p* < 0.001) differences were seen between the Alzheimeric rats and the free EGCG treated Alzheimeric rats, but no difference was seen between control rats and nanoEGCG treated Alzheimeric rats.

### NanoEGCG Attenuated the Generation of Aβ Plaques and Neurofibrillary Tangles in Alzheimeric Rat Brain Histopathology

**Figure 3:**
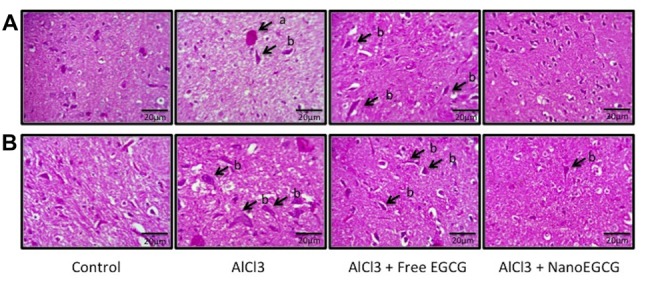
Representative histopathology images in the **(A)** cortex and **(B)** hippocampus regions of the rat brain (*n* = 3). Abbreviations: a, neuritic plaques and b, neurofibrillary tangles.

Sections of cortex and hippocampus tissues were stained with hematoxylin and eosin to study the pathological hallmarks of AD (Figure 3). The sections from control rats were intact with normal neuron and ganglion cell morphology with a normal appearing choroid plexus. Gross histopathological changes included the presence of amyloid plaques and neurofibrillary tangles. Alzheimeric rat brain sections showed the presence of scattered neurofibrillary tangles and scattered pyknotic cells and occasional eosinophilic amyloid plaques. Free EGCG treated Alzheimeric rat brain sections showed minimal perivascular inflammatory cell infiltration and few neurofibrillary tangles. However, in sections of nanoEGCG treated Alzheimeric rat brain sections showed normal cell architecture with normal appearing ganglion and neuronal cells with occasional areas showing a neurofibrillary tangle (Tables 1, 2).

**Table 1:** Effect of nanoparticles epigallocatechingallate (nanoEGCG) on the severity of histopathological alterations in the cortex of Alzheimeric rat brains.

Histopathological examinations	Control	AlCl_3_	AlCl_3_+Free EGCG	AlCl_3_+NanoEGCG
Degeneration & pyknosis in neurons	−	+++	+	−
Eosinophillic amyloid plaque formation	−	+	−	−
Neurofibrillary tangle formation	−	++	++	−

*Abbreviations: + mild, ++ moderate, +++ severe and −absent*.

**Table 2:** Effect of nanoEGCG on the severity of histopathological alterations in the hippocampus of Alzheimeric rat brains.

Histopathological examinations	Control	AlCl_3_	AlCl_3_+Free EGCG	AlCl_3_+NanoEGCG
Degeneration & pyknosis in neurons	−	+++	+	−
Eosinophillic amyloid plaque formation	−	−	−	−
Neurofibrillary tangle formation	−	+++	++	+

*Abbreviations: + mild, ++ moderate, +++ severe and −absent*.

### NanoEGCG Diminished Levels of Aβ_1–42_ in Alzheimeric Rats

**Figure 4:**
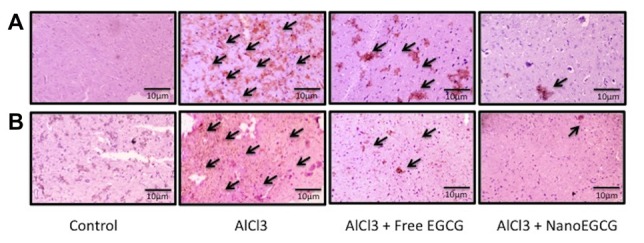
Representative immunohistochemical images in the **(A)** cortex and **(B)** hippocampus regions of the rat brain (*n* = 3). The arrows are indicative of Aβ_1–42_ levels.

**Figure 5:**
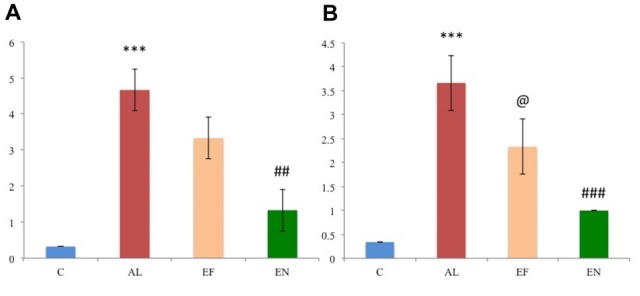
Scoring of the immunohistochemical images in the **(A)** cortex and **(B)** hippocampus regions of the rat brain (*n* = 3). Abbreviations: C, control; AL, AlCl_3_ induced Alzheimeric rats; EF, free EGCG treated AlCl_3_ induced Alzheimeric rats; EN, nanoEGCG treated AlCl_3_ induced Alzheimeric rats. Significance difference between groups indicated by: * between C and AL, ^@^ between AL and EF and ^#^ between AL and EN.Significance levels of *p* < 0.001, *p* < 0.01 or *p* < 0.05 are denoted by repetition of these symbols.

Sections of cortex and hippocampus tissues were subjected to immunochemical staining to study the distribution of Aβ_1–42_ (Figure 4). Control rat brain sections showed no Aβ_1–42_ levels. Alzheimeric rat brain sections showed significantly (*p* < 0.001) enhanced Aβ_1–42_ levels in both the cortex and hippocampus when compared to the control group. Treatment with free EGCG was able to marginally reduce the Aβ_1–42_ levels when compared to the Alzheimeric rats. However, treatment with nanoEGCG was able to drastically reduce the Aβ_1–42_ levels in both the cortex (*p* < 0.01) and hippocampus (*p* < 0.001) when compared to the Alzheimeric rats (Figure 5).

### NanoEGCG Reduced Acetylcholinesterase Activity in Alzheimeric Rats

**Figure 6:**
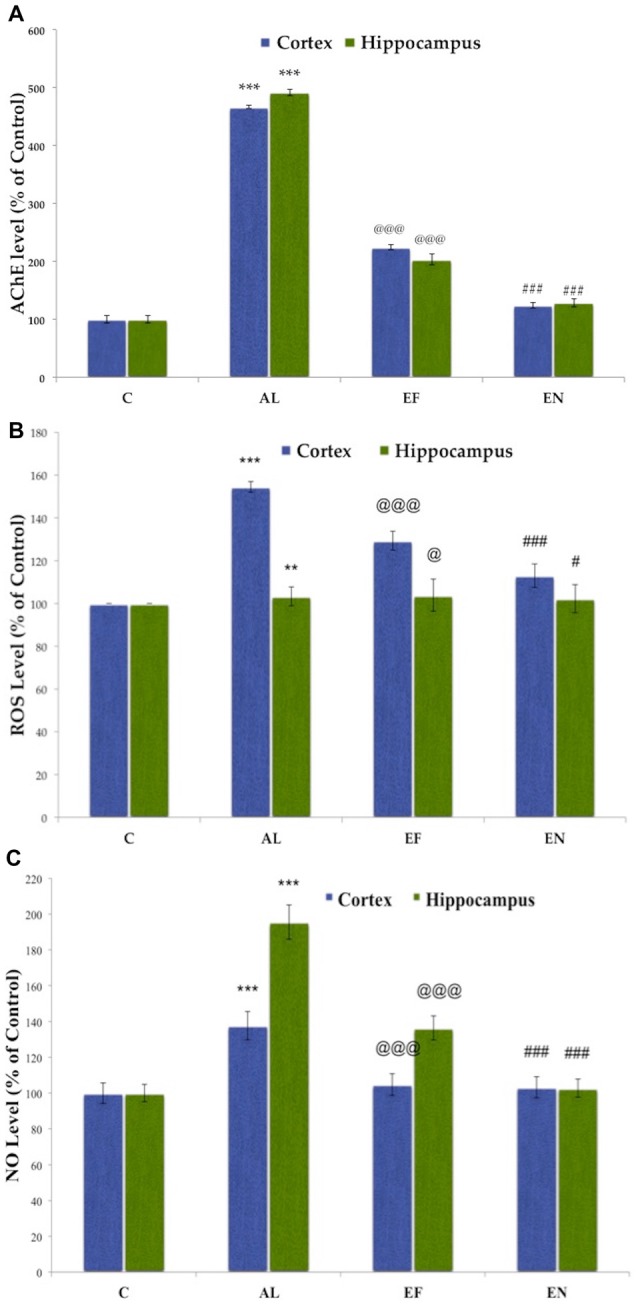
Effect of AlCl_3_ induction, free EGCG and nanoEGCG treatment on **(A)** Acetylcholinesterase activity, **(B)** Reactive oxygen species (ROS) generation and **(C)** Nitric oxide (NO) generation. Abbreviations: C, control; AL, AlCl_3_ induced Alzheimeric rats; EF, free EGCG treated AlCl_3_ induced Alzheimeric rats; EN, nanoEGCG treated AlCl_3_ induced Alzheimeric rats. Significance difference between groups indicated by: * between C and AL, ^@^ between AL and EF and ^#^ between AL and EN.Significance levels of *p* < 0.001, *p* < 0.01 or *p* < 0.05 are denoted by repetition of these symbols.

AChE is a marker for extensive cholinergic neuron loss in the brain. Alzheimeric rats showed significantly (*p* < 0.001) elevated levels of AChE in both the cortex and hippocampus when compared to the control rats. Free EGCG treatment of these Alzheimeric rats was able to significantly (*p* < 0.001) reduce these levels when compared to the Alzheimeric rats. However, nanoEGCG treated Alzheimeric rats showed that nanoEGCG is capable of significantly (*p* < 0.001) attenuating the AChE activity in both cortex and hippocampus when compared to the Alzheimeric rats (Figure 6A). No difference was seen between control rats and nanoEGCG treated Alzheimeric rats.

### Effect of NanoEGCG on the Biochemical Markers of Oxidative Stress in Alzheimeric Rats

#### NanoEGCG Attenuated the Elevated Levels of Reactive Oxygen Species

Elevated ROS production levels are markers of oxidative stress. A significant (*p* < 0.01) increase in ROS levels in the cortex and hippocampus has been observed in Alzheimeric rats when compared to control rats. Free EGCG treated Alzheimeric rats showed significantly (*p* < 0.05) lower ROS production levels, however, nanoEGCG treated Alzheimeric rats were able to significantly (*p* < 0.05) attenuate these ROS production levels in the rat brain when compared to Alzheimeric rats. Further, higher levels of ROS production were observed in the cortex rather than the hippocampus (Figure 6B).

#### NanoEGCG Attenuated the Elevated Levels of Nitric Oxide

Elevated NO production levels are markers of oxidative stress. A significant (*p* < 0.001) increase in NO levels in the cortex and hippocampus has been observed in Alzheimeric rats when compared to control rats. Free EGCG treated Alzheimeric rats showed significantly (*p* < 0.001) lower NO production levels, however, nanoEGCG treated Alzheimeric rats were able to significantly (*p* < 0.001) attenuate these NO production levels in the rat brain when compared to Alzheimeric rats. Further, higher levels of NO production were observed in the hippocampus rather than the cortex (Figure 6C).

### Effect of NanoEGCG on Protein Level Profiles for the Markers of Aβ Plaques and Neurofibrillary Tangles

**Figure 7:**
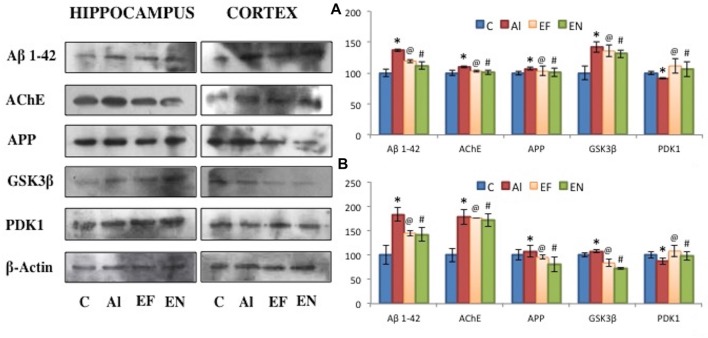
Effect of AlCl_3_ induction, free EGCG and nanoEGCG treatment on protein level profiles of Aβ_1–42_, AChE, APP, GSK3β and PDK1 in the **(A)** hippocampus and **(B)** cortex regions of the rat brain. Abbreviations: C, control; AL, AlCl_3_ induced Alzheimeric rats; EF, free EGCG treated AlCl_3_ induced Alzheimeric rats; EN, nanoEGCG treated AlCl_3_ induced Alzheimeric rats. Significance difference between groups indicated by: * between C and AL, ^@^ between AL and EF and ^#^ between AL and EN.

Protein level analysis was performed to investigate the changes in markers for Aβ plaques and neurofibrillary tangles (Figure 7). Alzheimeric rats showed a significant (*p* < 0.001) increase in levels of markers for Aβ plaques namely, Aβ_1–42_, AChE and APP in both cortex and hippocampus when compared to the control rats. A significant (*p* < 0.01) increase in levels of markers for neurofibrillary tangles namely, GSK3β and a significant decrease in PDK1 levels was noted in the cortex and hippocampus of Alzheimeric rats when compared to control rats. Further, on treatment with free EGCG significantly (*p* < 0.001) reduced levels of Aβ_1–42_, AChE, APP and GSK3β with significantly (*p* < 0.001) increased levels of PDK1 was noted when compared to Alzheimeric rats. However, when these Alzheimeric rats were treated with nanoEGCG significantly (*p* < 0.001) diminished levels of Aβ_1–42_, AChE, APP and GSK3β and significantly (*p* < 0.001) elevated level of PDK1 was observed in both the cortex and hippocampus when compared with Alzheimeric rats.

### Plasma and Brain Exposure of EGCG and Impact of EGCG Encapsulation in PLA-PEG Nanoparticles

**Figure 8:**
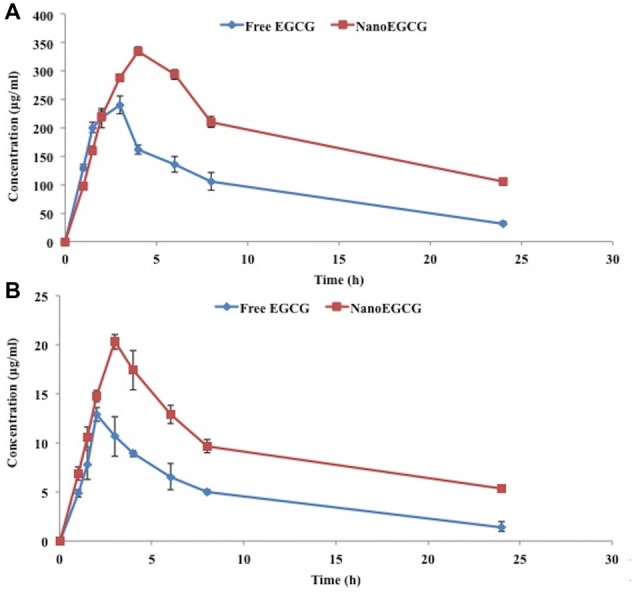
**(A)** Plasma and **(B)** brain concentration of unconjugated EGCG after the oral administration of free EGCG and nanoEGCG.

The bioavailability of nanoEGCG was evaluated by measuring the unconjugated EGCG in the plasma and brain (Figure 8). Oral administration of nanoEGCG showed a positive impact on the parameters for bioavailability. For unconjugated EGCG, in the plasma encapsulated EGCG had a maximum concentration of 334.65 ± 6.85 μg/ml, which took 4 h to achieve, when compared to free EGCG, which had a maximum concentration of 240.10 ± 15.30 μg/ml and took 3 h to achieve this concentration. In the brain encapsulated EGCG had a maximum concentration of 20.33 ± 0.75 μg/ml, which took 3 h to achieve, when compared to free EGCG, which had a maximum concentration of 12.90 ± 0.70 μg/ml and took 2 h to achieve this concentration (Table 3). Together this data suggest that encapsulation protects EGCG from degradation and increases its concentration in the plasma and brain. Moreover, the exposure over time (AUC_0-∞_) was higher for encapsulated EGCG i.e., 2,520.80 ± 66.70 mg.h/L in the plasma and 120.32 ± 7.50 mg.h/L in the brain when compared to free EGCG i.e., 1,103.29 ± 136.70 mg.h/L in the plasma and 51.92 ± 5.70 mg.h/L in the brain. This data further attests to higher bioavailability of nanoEGCG. Additionally, the clearance rate was also evaluated. For encapsulated EGCG the clearance rate was 0.022 ± 0.002 h^−1^ in the plasma and 0.036 ± 0.001 h^−1^ in the brain, which was slower when compared to free EGCG, which was 0.078 ± 0.006 h^−1^ in the plasma and 0.079 ± 0.016 h^−1^ in the brain. This further corroborates to nanoEGCG’s slow and sustained release.

**Table 3:** Bioavailability parameters of unconjugated epigallocatechingallate (EGCG) after oral administration of nanoEGCG.

Bioavailability parameters	Plasma		Brain	
	Free EGCG	NanoEGCG	Free EGCG	Nano EGCG
T_max_	3 h	4 h	2 h	3 h
C_max_	240.10 ± 15.3 μg/ml	334.65 ± 6.85 μg/ml	12.90 ± 0.7 μg/ml	20.33 ± 0.75 μg/ml
AUC_0-∞_	1103.29 ± 136.7 mg.h/L	2520.80 ± 66.7 mg.h/L	51.92 ± 5.7 mg.h/L	120.32 ± 7.5 mg.h/L

## Discussion

AD is a progressive neurodegenerative disease that deteriorates gradually over time (Pavlik et al., 2006). Aluminum accumulation has been insinuated to be a contributing agent to AD, where elevated aluminum concentrations in AD patient brains have been observed (Rondeau et al., 2009). It is said to enter the brain via transferrin like high affinity receptors and accumulate in the brain specifically in the hippocampus, the center for learning and memory (Roskams and Connor, 1990; Kaur and Gill, 2006). Aluminum has been reported to be a potent neurotoxicant, capable of inducing and accelerating brain oxidative damage, neuron death, cholinergic degradation, Aβ deposition, memory and learning deficits (Platt et al., 2001; Germano and Kinsella, 2005; Miu et al., 2006).

Our previous studies with nanoEGCG have resolved free EGCG’s bioavailability and stability issues. The fabricated nanoEGCG were size controlled and stable with a hydrodynamic diameter of 317.8 nm and an electrical charge of −24.5 mV. It had standard distribution with a polydispersity index of 0.115. NanoEGCG had a uniform, spherical to slightly ellipsoid shape and a smooth surface, with an encapsulation efficiency of 96.25 ± 1.01%. Here, the use of PVA as a surfactant maintained the stability and the size of the nanoparticles, while the PEG coating protected EGCG from the activity of gastrointestinal enzymes. *In vitro* Aβ_42_ fibrillation studies with nanoEGCG further attested to nanoEGCG’s ability to reduce neurotoxicity of Aβ_42_ by preventing the generation of toxic and insoluble Aβ_42_ fibrils. Hence, for the present study we chose to employ nanoEGCG as they offered high bioavailability and stability with controlled drug release, along with the ability to inhibitory effect on Al (III) induced Aβ_42_ fibrillation *in vitro* (Singh et al., 2018).

Results of the present study showed that chronic administration of AlCl_3_ led to the deterioration of working, spatial and recognition memory as determined by morris water maze and novel object recognition tests. Alzheimeric rats showed an increase in time taken to reach the platform and decrease in time spent in the target quadrant in the morris water maze. In case of novel object recognition test, Alzheimeric rats were unable to distinguish between the familiar and novel objects and were unable to recognize the novel object. Previous studies with chronic AlCl_3_ administration have shown similar results with learning deficits in both rats (Lakshmi et al., 2015) and rabbits (Rabe et al., 1982). These learning deficits and memory impairment together attest to aluminum’s ability to interfere with long-term memory potentiating molecules like cyclic GMP and produce an impairment in the glutamate-NO-cGMP pathway in the rat brain (Canales et al., 2001). NanoEGCG treatment was able to reverse the working, spatial and recognition memory deficits. Locomotion and exploratory patterns of Alzheimeric rats were also evaluated as a part of open field test. These rats showed a substantial locomotion and exploratory behavior impairment. Previous reports of chronic AlCl_3_ administration have described a decline in locomotion as well, which is implied to be an effect of central nervous system depression (Erazi et al., 2010). NanoEGCG treatment was able to correct the locomotion and exploratory behavior impairments. Together, these results attest to nanoEGCG’s neuroprotective role in correcting cognitive impairments.

Next, histopathological examinations of the different brain regions namely cortex and hippocampus was performed. Chronic administration of AlCl_3_ for 60 days showed the presence of eosinophilic amyloid plaques, scattered neurofibrillary tangles and scattered pyknotic cells along with neuronal degradation. NanoEGCG treatment was able to reverse this pathology considerably; this was shown by the complete absence of eosinophilic amyloid plaques with the presence an occasional neurofibrillary tangle. Immunohistochemical studies with Aβ_1–42_ were in cohort with the histopathology data. Here Alzheimeric rats showed significantly (*p* < 0.001) increased Aβ_1–42_ levels in both the cortex and hippocampus, while nanoEGCG treatment was able to attenuate immunoreactivity of Aβ_1–42_ substantially (*p* < 0.001). These results further demonstrate nanoEGCG’s ability to reverse the pathological hallmarks of AD and induce a protective effect on the cortex and hippocampal neuronal cells. The neurotoxicity generated by Aβ is said to trigger oxidative stress by the generation of ROS and NO. Further aluminum is a pro-oxidant that is known to cause oxidation in the presence of iron (Geremia et al., 1990). Previous studies with AlCl_3_ induced cognitive dysfunction models have reported elevation in levels of oxidative stress markers due to antioxidant enzyme system impairment (Khan et al., 2013). In the present study, Alzheimeric rats reported significantly elevated levels of ROS and NO. Treatment with nanoEGCG was able to attenuate both ROS and NO levels in the cortex and hippocampus and bring them back to normal when compared to the control rats.

Cholinergic transmission mainly affects learning, memory and cognition. It is closely related to short-term memory. The degree of impairment in this transmission correlates with the severity of dementia in patients (Amberla et al., 1993). Aluminum is a potent cholinotoxin (Gulya et al., 1990), which can alter the blood brain barrier to trigger changes in the cholinergic and noradrenergic transmission (Yokel, 2000). This neurotoxic effect of aluminum significantly elevates AChE activity, the key enzyme responsible for acetylcholine hydrolysis (Zatta et al., 2002). This elevation in AChE activity by aluminum could be due the interaction between aluminum and AChE peripheral sites that modify the secondary structure of AChE, thereby enhancing its activity (Kakkar and Kaur, 2011). The present study was in cohort with the previous literature, as here Alzheimeric rats showed considerably elevated AChE levels when compared to control rats. NanoEGCG treatment of these Alzheimeric rats was able to diminish these AChE levels drastically, possibly by reducing aluminum load in the brain. Therefore, nanoEGCG administration can potentially prevent cholinergic neuron loss in the brain.

Previous literatures on chronic AlCl_3_ administration have reported overexpression of APP (Luo et al., 2009), Aβ_1–42_, AChE (Ahmed et al., 2011) and GSK3β (Huang et al., 2017) levels, along with a reduction in the levels of PDK1 (Newington et al., 2012). In the present study, protein level studies reported a significant increase in APP, Aβ_1–42_, AChE and GSK3β with a significant decrease in PDK1 levels in Alzheimeric rats, when compared to control rats. This suggests that aluminum toxicity could be in favor of amyloid plaque and neurofibrillary tangle formation. Mechanism of action for this could be through the modulation of the PKC pathway, which is known to regulate the non-amyloidogenic α-secretase pathway (Cochran et al., 1990; Caporaso et al., 1992). NanoEGCG treatment was able to significantly reduce the levels of APP, Aβ_1–42_, AChE and GSK3β and elevate the levels of PDK1, when compared to the Alzheimeric rats. EGCG is known to induce its neuroprotective effect by binding with Aβ and prevent Aβ neurotoxicity by attenuating the free radicals (Ehrnhoefer et al., 2008). Further, EGCG is capable of activating the PKC pathway and reduce Aβ deposition. Additionally, EGCG can also activate the PI3K pathway, to prevent the hyperphosphorylation of GSK3β to inhibit neurofibrillary tangle generation. Moreover, EGCG is also capable of regulating tau pathology, by suppressing phosphorylated tau isoform (Singh et al., 2016). Therefore, nanoEGCG is capable of suppressing amyloid plaque and neurofibrillary tangle formation.

Bioavailability studies were carried to better understand PLA-PEG nanoparticles ability to increase free EGCG’s bioavailability. Previous literature on the subject has also suggested the same. Studies on rifampicin in gelatin nanocarriers in mice (Saraogi et al., 2010), mulberry 1-deoxynojirimycin in gelatin microspheres in rats (Vichasilp et al., 2012) and cryptolepine hydrochloride in gelatin nanoparticles (Kuntworbe et al., 2013) have all reported increase in drug concentration and retention time, when compared to the free drug. In the present study, nanoEGCG also showed higher drug concentration, retention time and AUC i.e., exposure over time when compared to free EGCG. This therefore, attests to nanoEGCG’s ability to enhance the bioavailability of EGCG in the plasma and brain.

## Conclusion

The present study clearly highlights nanoEGCG’s neuroprotective action against AlCl_3_ induced deficits. NanoEGCG was able to reverse AlCl_3_ induced neurobehavioral impairments and cognitive deficits including working, spatial and recognition memory loss, along with other locomotor impairments. It attenuated AlCl_3_ induced oxidative stress particularly ROS and NO activity and cholinergic deficits in both the cortex and hippocampus regions of the brain. Histopathological examinations demonstrated to nanoEGCG’s ability to reverse amyloid plaque and neurofibrillary tangle generation. Protein levels analysis for the markers of Aβ plaques and neurofibrillary tangles, further attested to nanoEGCG’s neuroprotective action against AD. Therefore, nanoEGCG can be considered as an effective therapeutic in the treatment of AD. Further explorations with transgenic mouse models and human clinical trials are needed to better understand the effects of nanoEGCG.

## Data Availability

All relevant data is contained within the manuscript. Videos for the neurobehavioral experiments are available on request.

## Author Contributions

AKAM and ZAK were responsible for concept and designing of this study. NAS performed the neurobehavioral experiments, immunohistochemical analysis, AChE assay and Western blot along with the analysis of the histopathological data. VB performed the oxidative stress assays. NR and CR performed and analyzed the bioavailability parameters. NAS was responsible for data analysis and drafting the article. AKAM and ZAK performed the final critical revision of the manuscript. All authors have read and approved the final manuscript.

## Conflict of Interest Statement

The authors declare that the research was conducted in the absence of any commercial or financial relationships that could be construed as a potential conflict of interest. The reviewer ESL and handling Editor declared their shared affiliation at the time of the review.

## Abbreviations

Aβ_42_: 1–42 amino acid form of beta amyloid
AChE: Acetylcholinesterase
AD: Alzheimer’s disease
AlCl_3_: aluminum chloride
Alzheimeric: AlCl_3_ induced Alzheimer disease
ANOVA: one-way analysis of variance
DTNB: 5,5′-dithiobis-(2-nitrobenzoic acid)
EGCG: epigallocatechin-gallate
H2DCFDA: dichlorodihydrofluorescein diacetate
HRP: horse-radish peroxidase
NanoEGCG: epigallocatechin-gallate loaded PLA-PEG nanoparticles
NO: Nitric oxide
P-tau: phosphorylated tau protein
PEG: polyethylene glycol
PLA: Poly (DL-lactic acid)
PVA: polyvinyl alcohol
ROS: Reactive oxygen species
TBST: Tris buffered saline—Tween 20 solution.
